# Acute ST-Elevation Myocardial Infarction Before and During the COVID-19 Pandemic: What is the Clinically Significant Difference?

**DOI:** 10.7759/cureus.10523

**Published:** 2020-09-18

**Authors:** Naeem Mengal, Tahir Saghir, Syed N Hassan Rizvi, Naveedullah Khan, Nadeem Qamar, Sobia Masood, Abida Badini

**Affiliations:** 1 Cardiology, National Institute of Cardiovascular Diseases, Karachi, PAK; 2 Research, National Institute of Cardiovascular Diseases, Karachi, PAK; 3 Family Medicine, Aga Khan Hospital for Women, Karachi, PAK

**Keywords:** acute st-elevation myocardial infarction, covid-19, primary percutaneous intervention, mortality

## Abstract

Background

In the current coronavirus disease-2019 (COVID-19) pandemic, the pattern of hospital admissions for acute ST-elevation myocardial infarction (STEMI) is changing, and increased mortality and morbidity is being noted in these patients. Cardiac manifestations of COVID-19 are complex and include STEMI, myocarditis, myocardial injury, and cardiomyopathy. The objective of our study was to compare the data of patients with STEMI presenting in COVID-19 versus the non-COVID-19 era.

Methods

We analyzed the clinical and angiographic characteristics of STEMI patients undergoing primary percutaneous coronary intervention (PCI) at our center. The primary outcome variables were admission rate for STEMI, mean total ischemic time (TIT), coronary artery disease burden, mean ejection fraction, and in-hospital mortality for three defined groups. Group A consisted of patients who underwent primary PCI from March through April 2020. Group B included patients who underwent primary PCI from January to February 2020. Group C consisted of patients who underwent primary PCI from March to April 2019. We then compared the data among the three groups and calculated any significant p-value (p<.001).

Results

In Group A, 1139 patients were admitted for primary PCI. The mean admission rate was 18.6 ± 4.36 admissions per day. There were 1535 patients in Group B and an admission rate of 26.01 ± 4.90 (p<.001 compared to Group A). In Group C, there were 1537 patients and an admission rate of 24.8 ± 4.55 (p<.001, compared to Group A). The mean TIT was 429.25±272.16 minutes for Group A, 359.78±148.04 minutes for Group B, and 346.75±207.31 minutes for Group C (p<.001). A higher mortality rate was noted in Group A (COVID-19 era) versus Group C (non-COVID-19 era; p<.001).

Conclusions

A lower admission rate, higher TIT, and higher mortality rates were noted in patients with acute STEMI during the COVID-19 pandemic compared to the pre-COVID era. During the COVID-19 pandemic, physicians should bear in mind that patients with STEMI have increased mortality and morbidity. Where possible, efforts should be made for timely management of these critical patients to decrease mortality.

## Introduction

Approximately more then 25 million cases of the coronavirus disease-2019 (COVID-19) and 844,312 deaths have been reported worldwide as of August 31, 2020 [1]. In Pakistan, the first case of COVID-19 was documented in late February 2020, and around three million confirmed cases and approximately 6294 mortalities have been reported [1].

In almost all parts of the world, social distancing and other measures are being practiced to prevent the spread of COVID-19; however, an increase in the admission rates of critically ill patients is still being noted [2-3].In this phase of the pandemic, the pattern of hospital admissions for acute ST-elevation myocardial infarction (STEMI) is also changing.

As COVID-19 prevalence grows exponentially, hospitals are being filled with patients while hospital supplies and medical staff are being exhausted. As a result, almost all elective procedures and surgeries have been postponed globally to manage COVID-19 patients [4].

Increased mortality and morbidity of cardiac patients have been noted in the COVID-19 era, but not all patients in need of cardiac care are infected with COVID-19. Data show that cardiovascular manifestations of COVID-19 are complex and variable, and patients may present with acute myocardial infarction (MI), myocarditis simulating a STEMI presentation, coronary spasm, myocardial injury, not fulfilling the criteria of Type 1 and Type 2 acute MI, and nonischemic cardiomyopathy. Conversely, patients with cardiovascular disease who develop COVID-19 have a higher risk of mortality [5-10]. Despite this, it is important to realize that not all patients who need care for the management of ischemic heart disease, peripheral vascular, or structural heart disease are infected with COVID-19. As we prepare for the care of patients with COVID-19, we should also make sure that patients with ischemic heart disease continue to benefit from the best available treatment [11-12]. Respiratory viral infections, including COVID-19, have been documented to increase cardiac complications such as STEMI (acute coronary occlusion) and myocarditis [6-13].

COVID-19 is a new disease, and as new data are emerging, information about the natural history, clinical presentation, and involvement of different organ systems is being evaluated. In this study, we have tried to determine some important differences among patients presenting with STEMI during the COVID-19 pandemic versus the non-COVID-19 era. It will help the clinicians to better understand these patients and plan timely management.

## Materials and methods

We analyzed the clinical and angiographic characteristics of STEMI patients undergoing primary PCI at the National Institute of Cardiovascular Diseases (NICVD) in Karachi, Pakistan. The NICVD in Karachi is the largest tertiary care cardiac hospital in the country. Along with its 10 satellite centers for the treatment of STEMI patients with primary PCI as the only treatment strategy, NICVD performs more than 28,000 procedures per year. In Pakistan, the first case of COVID-19 was documented on February 26, 2020; therefore, we defined our study period for two months from March 1, 2020, to April 30, 2020 and compared it with the non-COVID-19 era.

Baseline characteristics of patients were obtained. We then calculated the primary outcome variables; the overall hospital admissions rate for STEMI patients undergoing primary PCI; mean total ischemic time (TIT) (calculated from the onset of symptoms to the first balloon inflation in minutes); coronary artery disease burden (number of coronary vessels diseased); mean ejection fraction, which was derived from left ventriculogram on right anterior oblique (RAO) 30° projection; and in-hospital mortality (defined as death during the hospital stay) for the three defined groups.

Group A consisted of patients who underwent primary PCI during the study period from March 1, 2020 to April 30, 2020, which encompassed two months of the COVID-19 pandemic. Group B consisted of patients who underwent primary PCI two months prior, from January through February of 2020. Group C consisted of patients who underwent primary PCI during the same period in the previous year (March 1, 2019 to April 30, 2019).

The clinical characteristics of the patients were compared among the three groups. Quantitative comparison was made by applying an appropriate independent sample t-test or Mann-Whitney U test, and a categorical comparison was made by applying the Chi-square/Fisher's exact test and calculating any significant p-value (p<0.001). A multivariate analysis was conducted to exclude any confounding factors.

## Results

In Group A, 1139 patients with acute STEMI who underwent primary PCI were admitted. Eighty percent (911) were men; the mean age (±SD) was 55.5 ± 11.5 years, and the mean admission rate was 18.6 ± 4.36 admissions per day. There were 1535 patients in Group B with an admission rate of 26.01 ± 4.90 per day (p<.001) and 1537 patients in Group C with an admission rate of 24.8 ± 4.55 per day (p<.001).

**Figure 1:**
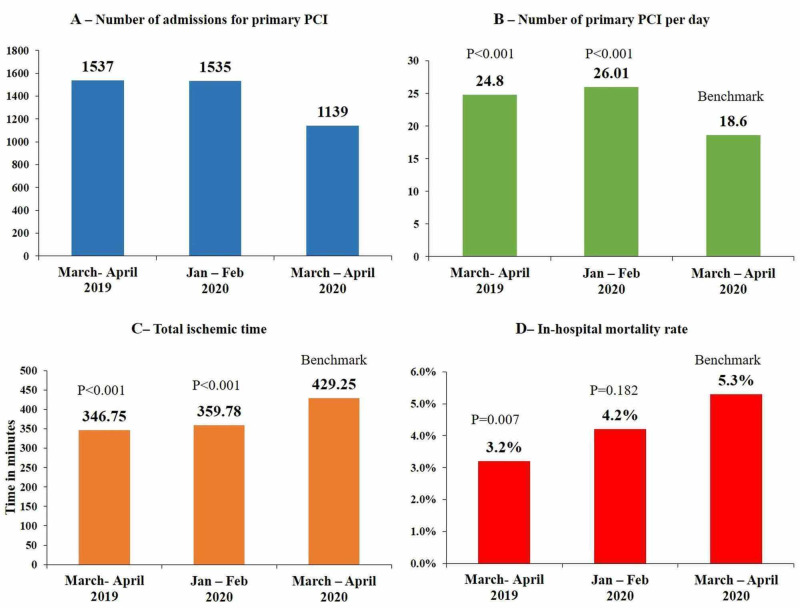
Significant differences in clinical characteristics PCI, percutaneous coronary intervention

Patients with hypertension totaled 472 (41.4%), 596 (38.8%), and 611 (39.7%) in Groups A, B, and C, respectively. There were approximately 396 (34.7%), 539 (35.1%), and 521 (33.8%) patients with diabetes in Groups A, B, and C, respectively. Additionally, 282 (24.7%), 403 (26.2%), and 411 (26.7%) patients identified as smokers in the above three corresponding groups. The mean TIT was 429.25±272.16 minutes for Group A, 359.78±148.04 minutes for Group B, and 346.75±207.31 minutes for Group C. A significant p-value (p<.001) was noted compared to the reference group. Mean ejection fraction (derived from left ventriculogram in plain RAO 30° projection) was 34.74±18.16 for Group A, 31.58±19.15 for Group B (p<.001), and 36.14±16.89 for Group C. No significant difference was noted among the three groups regarding gender distribution and burden of coronary artery disease. Of important note was the statistically significant (p<.001) mortality rate in Group A (5.3%) as compared to Group C (Table 1) (Figure 1). In the COVID-19 era, 25 of our patients were COVID-19 positive, and six patients had a Type 2 acute MI. The workup for COVID-19 in these patients was due to the associated typical signs and symptoms of infection.

**Table 1:** Clinical characteristics of the study population STEMI, ST-elevation myocardial infarction; HTN, hypertension; DM, diabetes mellitus; EF, ejection fraction; CAD, coronary artery disease; SVCAD, single vessel coronary artery disease; DVCAD, double vessel coronary artery disease; TVCAD, triple vessel coronary artery disease; LM, left main coronary artery

Variables	Group A	Group B	Group C
	March – April 2020	Jan – Feb 2020	March- April 2019
Total no. of patients	1139	1535	1537
Average no. of Admissions (STEMI) / day	18.6 ± 4.36*	26.01 ± 4.90(p<0.001)	24.8 ± 4.55(p<0.001)
Mean Age (years)	55.52±11.54*	55.98±12.05(p=0.320)	52.72±13.80(p<0.001)
Male gender(%)	80.7*	78.4(p=0.146)	77.2(p=0.029)
HTN (%)	472 (41.4)	596 (38.8)	611 (39.7)
DM (%)	396 (34.7)	539 (35.1)	521 (33.8)
Smoking (%)	282 (24.7)	403 (26.2)	411 (26.7)
Mean Total ischemic time (min)	429.25±272.16*	359.78±148.04(p<0.001)	346.75±207.31(p<0.001)
Mean EF(%)	34.74±18.16*	31.58±19.15(p<0.001)	36.14±16.89(p=0.040)
CAD Burden (%)			
SVCAD	37.5	35.0	35.7
DVCAD	30.6	32.7	33.8
TVCAD	28.5	29.3	27.3
LM±SV/DV/TVCAD	3.4	2.9	3.2
Mortality(%)	5.3*	4.1(p=0.182)	3.2(p=0.007)

## Discussion

The most common presentations of COVID-19 are that of respiratory symptoms, and cardiovascular involvement can be the initial presentation or can complicate the course of the disease. Mortality and morbidity are very high in these patients [14].

Several mechanisms have been explained by which COVID-19 can involve the cardiovascular system. It causes direct MI by altering angiotensin-converting enzyme 2 (ACE2) signaling pathways as it binds to ACE2 receptors of the myocardium and lungs [15-16]. Hypoxia and respiratory failure create a demand-supply mismatch and, as a result, cause acute MI (Type 2 acute MI). Additionally, systemic inflammatory response syndrome and the release of cytokines further aggravate the situation and result in multi-organ failure and circulatory collapse [17-18]. Multi-organ failure resulting in a major electrolyte imbalance puts the patient at risk for arrhythmias. The most common cardiovascular complication of COVID-19 is acute MI, seen in approximately 8% to 12% of COVID-19-related cases [19]. Direct myocardial involvement of the virus was also noted in autopsies during the severe acute respiratory syndrome (SARS) outbreak in 2003 [20].

Regardless of COVID-19 status, in patients with STEMI, the Society for Cardiovascular Angiography and Interventions (SCAI) recommends adopting an invasive strategy where indicated [4]. Different reasons for delayed cardiac catheterization laboratory (CCL) activation have been postulated in the COVID-19 pandemic. In Hong Kong, a study showed delays in symptom onset to first medical contact time in STEMI patients during the COVID-19 outbreak. The study also suggests other patients- and/or systems-related delays in medical care provision during this pandemic [10]. A study from the United States shows a 38% reduction in CCL activation for STEMI patients in the early days of the COVID-19 pandemic. Initially, an increase in STEMI CCL activation was expected, considering the potential heightened environment and fear. Potential reasons for the subsequent decrease in STEMI PCI CCL activations could include the avoidance of medical care due to social distancing or concerns of contracting COVID-19 in the hospital, STEMI misdiagnosis given the focus on respiratory issues, and increased use of pharmacological reperfusion due to COVID-19 [21].

Patient COVID-19 status can be evaluated via a focused history. A more detailed evaluation may be needed in some cases in the ED in case of suspected cases but that can be another reason for longer door-to-balloon times during the COVID-19 pandemic. Myocarditis and stress cardiomyopathy have also been noted in association with COVID-19 and may mimic STEMI [22,23].

Our study shows a significant decline of about 27% in all STEMI admissions in the largest tertiary care cardiac hospital in Pakistan, during the first two months of the COVID-19 pandemic. Studies report that fewer STEMI patients have been seeking care during the pandemic. There are concerns that these patients are coming later to the ED or not coming at all due to the fear of exposure to patients with COVID-19 [4]. These STEMI patients will suffer unnecessary morbidity and mortality without proper management. Effort should be made to convince these patients that, in case of concerns suggestive of an acute coronary syndrome (ACS), they should come in for proper evaluation while ensuring appropriate screening and protection from severe acute respiratory syndrome coronavirus 2 (SARS-CoV-2) infection [4]. There is a possibility that some patients might have died due to STEMI without seeking cardiac care because of this pandemic, decreasing the rate of hospital admissions.

As discussed above, in the United States, an approximate 38% reduction in CCL STEMI activations has been noted during the COVID-19 pandemic [21]. Initially, it was expected that CCL activation might increase STEMI, induced by stressors and viral illness that mimics STEMI, such as myopericarditis. Potential reasons for the decrease in STEMI CCL activations could include avoidance of medical care due to social distancing or concerns of contracting COVID-19 in the hospital; STEMI misdiagnosis, given the focus on respiratory issues; and increased use of pharmacological reperfusion due to COVID-19 [4].

Another important finding in the study data was a significant increase in the TIT (i.e., the duration from the onset of symptoms to the first device activation in CCL). An increase in TIT can increase mortality. Determinants of TIT are multifactorial, including patient inhibition and fear, time delays due to unavailability of emergency medical services (EMS), delays due to evaluation of patients in the ED to exclude any suspected COVID-19 diagnosis, and then activation of CCL.

Of note in this study, is the significant increase in the mortality in the COVID-19 era, which was consistent after multivariate analysis. This increase in mortality rate is not fully explainable by COVID-19 status alone as, out of 1139 patients, there were only 25 COVID-19 positive patients. Of these 25 patients, six had Type 2 acute MI. All laboratory procedures were performed in full personal protective equipment irrespective of COVID-19 status. The workup for COVID-19 in these patients was due to typical signs and symptoms. The facility did not provide rapid testing for every STEMI patient in the ED before shifting to CCL; however, there was a dedicated CCL for COVID-19 positive patients or suspected cases. A sample for COVID-19 testing was sent after the primary PCI in all suspected cases. A dedicated floor for any positive or suspected COVID-19 case was run by a dedicated infectious disease critical care team in collaboration with a cardiologist. As the majority of COVID-19 cases are asymptomatic, they could have increased mortality without being noticed.

The Chinese Center for Disease Control and Prevention reported that in 72,314 cases (with 44,672 COVID-19 confirmed) the crude mortality rate was 2.3%. The case fatality rate was 14.8% in octogenarians. Coronary heart disease (CHD) was present in 4.2% of all cases but was 22.7% in fatal cases. Case fatality rates were 10.5% in patients with CHD, 7.3% in patients with diabetes, and 6% in patients with hypertension [24].

Because of the COVID-19 pandemic, regional STEMI care and systems are changing, and interventional cardiology teams are working under high stress. A workable network is needed among EMS, referral hospitals, and primary PCI centers. SCAI recommends that these entities of STEMI care should revise their plan of action according to the local needs to minimize the delay in patient care. Additional noninvasive evaluation in the ED is recommended for patients with an unclear diagnosis of STEMI, diffuse ST-segment elevation, atypical history or electrocardiogram findings, or a delayed presentation. A point of care ultrasound of the heart or a transthoracic echocardiographic evaluation can be performed for evaluation of any wall motion abnormality consistent with electrocardiographic changes. Coronary computed tomography angiography can also be considered under challenging scenarios [4].

## Conclusions

Our data show a low admission rate with higher TIT and mortality in STEMI patients during the COVID-19 pandemic. COVID-19 is a new disease, and as new data are emerging, information about the natural history, clinical presentation, and involvement of different organ systems is being evaluated. Cardiac involvement is variable and includes ACS. Patients with known heart disease and COVID-19 are at a higher risk of mortality. Patients with STEMI should be given standard care of primary PCI with an effort to minimize TIT and mortality.
